# The Phylogeny and Evolutionary Timescale of Muscoidea (Diptera: Brachycera: Calyptratae) Inferred from Mitochondrial Genomes

**DOI:** 10.1371/journal.pone.0134170

**Published:** 2015-07-30

**Authors:** Shuangmei Ding, Xuankun Li, Ning Wang, Stephen L. Cameron, Meng Mao, Yuyu Wang, Yuqiang Xi, Ding Yang

**Affiliations:** 1 Department of Entomology, China Agricultural University, Beijing, China; 2 Institute of Grassland Research, Chinese Academy of Agricultural Sciences, Hohhot, China; 3 Earth, Environmental and Biological Sciences School, Science & Engineering Faculty, Queensland University of Technology, Brisbane, Australia; 4 Centre for Medical Bioscience, School of Biological Sciences, University of Wollongong, Wollongong, NSW 2522, Australia; Sichuan University, CHINA

## Abstract

Muscoidea is a significant dipteran clade that includes house flies (Family Muscidae), latrine flies (F. Fannidae), dung flies (F. Scathophagidae) and root maggot flies (F. Anthomyiidae). It is comprised of approximately 7000 described species. The monophyly of the Muscoidea and the precise relationships of muscoids to the closest superfamily the Oestroidea (blow flies, flesh flies etc) are both unresolved. Until now mitochondrial (mt) genomes were available for only two of the four muscoid families precluding a thorough test of phylogenetic relationships using this data source. Here we present the first two mt genomes for the families Fanniidae (*Euryomma* sp.) (family Fanniidae) and Anthomyiidae (*Delia platura* (Meigen, 1826)). We also conducted phylogenetic analyses containing of these newly sequenced mt genomes plus 15 other species representative of dipteran diversity to address the internal relationship of Muscoidea and its systematic position. Both maximum-likelihood and Bayesian analyses suggested that Muscoidea was not a monophyletic group with the relationship: (Fanniidae + Muscidae) + ((Anthomyiidae + Scathophagidae) + (Calliphoridae + Sarcophagidae)), supported by the majority of analysed datasets. This also infers that Oestroidea was paraphyletic in the majority of analyses. Divergence time estimation suggested that the earliest split within the Calyptratae, separating (Tachinidae + Oestridae) from the remaining families, occurred in the Early Eocene. The main divergence within the paraphyletic muscoidea grade was between Fanniidae + Muscidae and the lineage ((Anthomyiidae + Scathophagidae) + (Calliphoridae + Sarcophagidae)) which occurred in the Late Eocene.

## Introduction

Muscoidea, with approximately 7000 described species, is nearly 5% of the known species-level diversity of the Diptera, the true flies [1], and includes the following four families: Scathophagidae, Anthomyiidae, Fanniidae and Muscidae. Most muscoid flies are saprophagous, coprophagous or necrophagous as larvae, but some species are parasitic, predatory or phytophagous. Besides these ‘positive’ roles in the ecosystem, some species such as houseflies carry various pathogenic microorganisms (e.g. *Escherichia coli*, *Shigella* spp., *Salmonella* spp.) [2].

Although muscoid flies have economic and medical importance, with high ecological significance, their phylogenetic relationships are still controversial. Numerous phylogenetic studies have been carried out with morphological and molecular data to reconstruct relationships either within Muscoidea or more broadly across the Calyptratae [1, 3–5]. McAlpine (1989) recovered Muscoidea as a monophyletic group based on a combination of morphological character states [3], while Michelsen (1991) pointed out that Muscoidea was considered as ‘‘the Calyptratae less the Hippoboscoidea and Oestroidea” [6]. Based on four mitochondrial and four nuclear genes from 127 exemplar species of Muscoidea, Kutty et al. (2008) found that Muscoidea was paraphyletic, with the clade (Anthomyiidae + Scathophagidae) sister to a monophyletic Oestroidea. This set of relationships was subsequently also found by Kutty et al. (2010) and Wiegmann et al. (2011) in molecular analyses utilizing different taxon and gene combinations from thosed used in Kutty et al. (2008). On the other hand, in the most comprehensive morphological phylogenetic analysis of flies ever conducted, Lambkin et al. (2013) recovered the clade Scathophagidae + (Anthomyiidae + (Muscidae + Oestroidea)) [7]; Fanniidae was not included in this analysis. The evolutionary age of the Muscoidea has also never been directly assessed by phylogenetic means. Wiegmann et al. (2003) [8] suggested that the Calyptratae (represented by a muscid) diverged from the acalyptrates (represented by a drosophilid) between 50 and 80 million years ago (MYA), although the age confidence intervals for this split ranged from 5–130 MYA. In a subsequent, more intensive analysis, Wiegmann et al. (2011) [5] inferred an age of 50 MYA for the clade Muscoidea+Oestroidea, though no confidence interval was included. The age of splits within the Muscoidea has yet to be examined.

Mitochondrial (mt) genomes have been established as a powerful tool for reconstructing phylogenetic relationships because of their ability to provide more phylogenetic information than individual genes [9–16]. The use of whole mt genome sequences in insect phylogenetics has produced some remarkable results, e.g., the polyphyly of Hexapoda [17] and the monophyly of Megaloptera [18]. Cameron et al. (2014) [19] reviewed the use of mt genomic data in insect phylogenetics and provided guidelines for the testing for analytical biases introduced by base compositional bias, rate variation and partitioning schemes.

Since Clary and Wolstenholme published the first insect mitochondrial genome sequence in 1985 [20], Diptera has been a primary model system for mt genome research both for phylogenetic and molecular evolution studies. The number of mt genome sequences from Diptera deposited in GenBank has grown rapidly. As of May 2015, 111 complete or nearly complete dipteran mt genomes had been published on GenBank, representing 29 families. However, much of the dipteran mt genome data is derived from species of the model organism *Drosophila* [21], whereas most families have not been sequenced and those families for which mt genomes are available are represented by only one or two species. This is particularly true for the muscoid families. Prior to this study, there were only four complete mt genome sequences available for Muscoidea, representing two of the four families: Muscidae (from which three species have been sequenced) and Scathophagidae (one species sequenced).

Herein, we presented two nearly complete mt genomes representing the remaining two muscoid families: *Euryomma* sp. (Fanniidae) and *Delia platura* (Meigen, 1826) (Anthomyiidae). We used procedures and quality control methods proposed by Cameron [22] to re-annotate all mt genomes from muscoid flies and examine genome variability across the group. A phylogenetic analysis of cyclorrhaphan mt genomes finds further evidence that Muscoidea is paraphyletic, which a molecular dating analysis suggests that divergence of the muscoid grade occurred in the Eocene.

## Material and Methods

### Ethics statement

No specific permits were required for the insects collected for this study. The specimens were collected using sweep nets. The field studies did not involve endangered or protected species. The species studied herein are not included in the “List of Protected Animals in China”.

### Specimen Collection, DNA Extraction

The *Euryomma* sp. specimen used for DNA extraction was collected by Jinying Yang and Lei Zhang from Gaoshang, Jining, Shandong, China (N35°05′0.94″ E116°19′34.92″) in May 2013, the *Delia platura* specimen was collected by Yuqiang Xi from Huangniupu, Fengxian, Shaanxi, China (N34°11′47.11″ E106°49′34.12″) in August 2013. After collection, they were initially preserved in 95% ethanol in the field, and transferred to -20°C for the long-term storage upon the arrival at the China Agricultural University (CAU). The specimens were examined and identified by the corresponding author Ding Yang with a ZEISS Stemi 2000–c microscope. Total DNA was purified from muscle tissues of the thorax using TIANamp Genomic DNA Kit (TIANGEN). The quality of DNA was assessed through electrophoresis in a 1% agarose gel and stained with Goldview nucleic acid stain (Guangzhou Geneshun Biotech, Guangdong China).

### PCR Amplification and Sequencing

The mt genome was generated by amplification of 20 overlapping PCR fragments using NEB Long Taq DNA polymerase (New England BioLabs, Ipswich, MA). Initial fragments were amplified using universal primers [15]. Several species-specific primers were designed based on results from initial sequencing with amplification primers and used for internal PCRs (S1 Table). The amplification conditions were: hotstart denaturation at 95°C for 30 sec, 40 cycles of denaturation at 95°C for 10 sed, primer annealing at 40–48°C for 50 sec and extension at 65°C for 1 min/kb expected size of amplicons, and the final elongation step at 65°C for 10 min. The quality of PCR products were evaluated by agarose gel electrophoresis. All fragments were sequenced in both directions using the ABI BigDye Terminator Sequencing Kit ver. 3.1 (Applied Bio Systems) using amplification and internal primers for primer walking and products run on the ABI 3730XL Genetic Analyzer (PE Applied Biosystems, San Francisco, CA, USA).

### Sequence Assembly and Annotation

Mt DNA sequences were proof-read and aligned into contigs using BioEdit ver. 7.0.5.3 [23]. Sequence analysis was performed as follows. First, the tRNA genes were identified by tRNAscan-SE Search Server v.1.21 using invertebrate mitochondrial predictors with a COVE cutoff score of 1, or by sequence similarity to tRNAs of other Diptera. PCGs were identified as open reading frames corresponding to the 13 PCGs found in metazoan mt genomes. The rRNA gene boundaries were interpreted as the end of a bounding tRNA gene and by alignment with other Diptera gene sequences. Base composition, codon usage, and nucleotide substitution statistics were analyzed using MEGA ver. 5.0 [24]. Stand compositional asymmetry was measured in terms of GC and AT skews using the following formulae: AT-skew = (A-T)/(A+T) and GC-skew = (G-C)/(G+C) [25]. Secondary structures of the small and large subunits *rRNA* were inferred using models predicted for *Drosophila yakuba* (Diptera) [20], *Apis mellifera* (Hymenoptera) [26], and *Libelloides macaronius* (Neuroptera) [27].

### Phylogenetic Analysis

Phylogenetic analysis was conducted on a taxon set of 17 species of Brachycera, including 15 cyclorrhaphans and 2 outgroup species from the families Tabanidae and Nemestrinidae, which belong to the basal Brachycera and non-heteroneuran muscomorphs respectively. Mt genomes for 15 species were downloaded from GenBank, and two species of Muscoidea (Table 1) are newly sequenced for this study.

**Table 1:** Taxon sampling and availability.

**Family**	Species	Published information	GenBank Accession No.	Length (bp)
Tabanidae	*Trichophthalma punctata#*	[28]	NC_008755	16396
Nemestrinidae	*Cydistomyia duplonotata#*	[28]	NC_008756	16247
Phoridae	*Megaselia scalaris*	Zhong et al. unpublished	NC_023794	15599
Syrphidae	*Simosyrphus grandicornis*	[28]	NC_008754	16141
Drosophilidae	*Drosophila melanogaster*	[29]	NC_001709	19517
	*Drosophila santomea*	[30]	NC_023825	16022
	*Drosophila yakuba*	[20]	NC_001322	16019
Oestridae	*Hypoderma lineatum*	[31]	NC_013932	16354
Tachinidae	*Elodia flavipalpis*	[32]	NC_018118	14932
Calliphoridae	*Chrysomya putoria*	[33]	NC_002697	15837
Sarcophagidae	*Sarcophaga impatiens*	[34]	NC_017605	15169
Scathophagidae	*Scathophaga stercoraria*	[35]	NC_024856	16223
Muscidae	*Musca domestica*	[35]	NC_024855	16108
	*Stomoxys calcitrans*	[36]	DQ533708	15790
	*Haematobia irritans*	Lessinger et al. unpublished	NC_007102	16078
Anthomyiidae	*Delia platura*			15315+
Fanniidae	*Euryomma* sp.			14858+

Note: “#” outgroup.

All three mt gene classes were used in this phylogenetic analysis, the 13 PCGs, 2 rRNAs and 22 tRNAs. Each PCG was aligned individually using ClustalW [37] and gene annotations checked using the procedures proposed by Cameron (2014) [22]. The tRNA and rRNA genes were aligned using ClustalW as implemented in MEGA 5.0 [24]. Ambiguously aligned positions in the RNA alignments were removed by hand. Individual gene alignments were concatenated using SequenceMatrixv1.7.8 [38]. We assembled four datasets for phylogenetic analysis: 1) the 13 PCGs with all three codon positions included (PCG123) (11,157bp), 2) the 13 PCGs (all codon positions), plus the two rRNAs and 22 tRNAs (PCG123RNA) (13,325bp), 3) the 13 PCGs alone, excluding third codon positions (PCG12) (7,438bp) and 4) the 13 PCGs (excluding third codon positions), plus the 2 rRNAs and 22 tRNAs (PCG12RNA) (9,606bp) (see S3 Table). PartitionFinder v1.1.1 [39] was used to select the optimal partition strategy and substitution models for each partition. We created an input configuration file with 63 or 50 (with vs.without 3rd codon positions) pre-defined partitions (1 for each codon position of each PCG plus 1 for each rRNA or tRNA gene), and used the ‘‘greedy” algorithm with branch lengths estimated as ‘‘unlinked” and Bayesian information criterion (BIC) to search for the best-fit scheme. Optimal partitioning scheme and associated models for each of the 4 datasets outlined above are given in S3 Table.

We performed maximum likelihood (ML) and Bayesian-inference (BI) analyses using the best-fit partitioning schemes recommended by PartitionFinder. For ML analysis, we used RAxML 8.0.0 [40] with 1,000 bootstrap replicates and the rapid bootstrap feature (random seed value 12345) [41]. Bayesian analysis was conducted with MrBayes 3.2.2 [42]. Two simultaneous runs of 2 million generations were conducted for the datasets, each with one cold and three heated chains. Samples were drawn every 1,000 Markov chain Monte Carlo (MCMC) steps, with the first 25% discarded as burn-in. When the average standard deviation of split frequencies was below 0.01, we considered that stationarity was reached and stopped the run.

### Estimation of Divergence Time

Divergence times were estimated using the Bayesian Markov Chain Monte Carlo approach (MCMC) implemented in the software in BEAST v.1.8.0 [43]. We simultaneously inferred topology and node ages by estimating the Bayesian posterior distribution of divergences. The uncorrelated, relaxed lognormal clock was applied to model rate variation among lineages [44]. The *ucld*.*stdev* parameter was close to 0 in all partitions, meaning that the data were clock-like and that the rate of heterogeneity among species was low [44]. The GTR substitution mode and empirical base frequencies were applied as a Tree prior. The Yule speciation tree prior was chosen to calculate the divergence times among taxa. Based on previous research on divergence times in Diptera [5, 8, 45–47], we implemented fossil-based age constraints for three clades, 195 mya for Brachycera, 125 mya for Cyclorrhapha and 64 mya for Schizophora [48–49]. Analyses consisted of three separate MCMC runs each of 10 million generations sampled every 1000 steps. Tracer v1.5 [50] was used to check the effective sample sizes of all the parameters which were greater than 100. Finally, the maximum-clade-credibility tree was calculated using TreeAnnotator v1.4.8 [51]. FigTree v1.4 [52] was employed for visualization of the tree.

## Results and Discussion

### General features of Fanniidae and Anthomyiidae mt genomes

**Fig 1:**
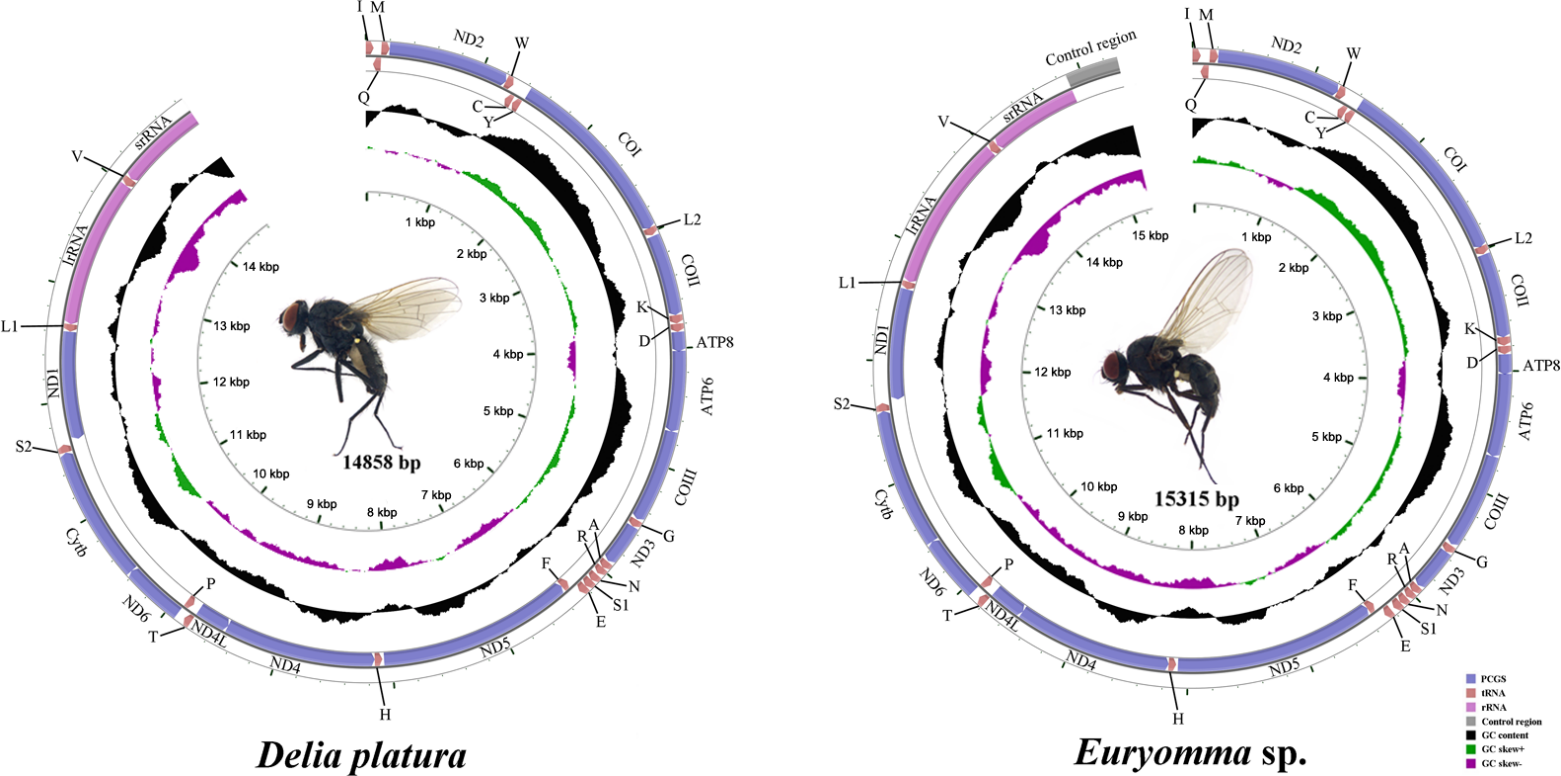
Mitochondrial maps of two muscoid flies. Circular maps were drawn with GCView [53] with the unsequenced portion of each genome indicated. Gene arrangement is shown on the outermost circle with arrows indicating the orientation of gene transcription. tRNAs are are labelled according to the IUPACIUB single-letter amino acid codes (L1: CUN; L2: UUR; S1: AGN; S2: UCN). GC content was plotted (in black) using a sliding window, as the deviation from the average GC content of the entire sequence. GC-skew was plotted (positive skew in green, negative skew in purple) as the deviation from the average GC-skew of the entire sequence. The innermost cycle indicates size.

The near complete mt genomes of *Euryomma* sp. (Fannidae) and *Delia platura* (Anthomyiidae) were sequenced. The lengths of mt genomes among Diptera, range from 14,922 bp in *Sarcophaga peregrine* (Sarcophagidae, Zhong et al., unpublished; control region is 123 bp in length) to 19,517 bp in *Drosophila melanogaster* (Drosophilidae [29]; CR 4601 bp long), with both newly sequenced species in the middle of this range. The mt genomes of muscoid flies have the typical circular, double-stranded molecule (GenBank accession number: KP01268, KP01269; Fig 1) with length of about 16000 bp, and contain 37 genes including 13 PCGs, 22 tRNA genes, 2 rRNA genes and a control region, found in almost all bilaterian animals [22]. The gene order in both species is the same as that of the ancestral insect mt genome (as in almost all Diptera), with 23 genes encoded on the majority strand (J-strand), while the minority strand (N-strand) encodes the remaining 14 genes. Some general characteristics of the genomes are given in S4 Table and S5 Table. The near complete mt genomes of *Euryomma* and *Delia* are 15315 bp and 14858 bp in length respectively (Fig 1), however we failed to complete sequencing of their control regions. Genome annotation statistics are listed in S4 and S5 Tables.

The *Euryomma* mt genome has 10 regions of overlap between genes, ranging in size from 1 to 7 bp (S4 Table). The longest is between *ATP8* and *ATP6* and has been found in many other insect species from multiple orders. In other muscoid species, however, the longest gene overlap region is between *tRNA* ^*Trp*^ and *tRNA* ^*Cys*^ and is 8 bp long. Fifteen intergenic spacers, ranging in size from 1 to 27 bp, were identified in the *Euryomma* mt genome. The longest includes a microsatellite-like TA_n_ repeat (GATATAAATTATATATATATATATATA), located between *tRNA* ^*Ser(AGN)*^ and *tRNA* ^*Glu*^. By contrast, the longest intergenic spacer found in the mt genomes of other muscoid species is consistently the one located between *tRNA* ^*Glu*^ and *tRNA* ^*Phe*^ and is size invariant (18 bp) across the superfamily, and also conserved among the Cyclorrhapha ranging in size from 65 bp (*Fergusonina taylori*, Fergusoninidae, [34]) to 16 bp (*Sarcophaga peregrine*, Sarcophagidae, Zhong et al., unpublished) [54]. In the mt genome of *Delia*, 10 overlapping regions ranging from 1 to 8 bp in size and 13 intergenic spacers ranging from 1 to 18 bp in size were detected (S5 Table). The longest gene overlap region is between *tRNA* ^*Trp*^ and *tRNA* ^*Cys*^ and the longest intergenic spacer is between *tRNA* ^*Glu*^ and *tRNA* ^*Phe*^; both are conserved across muscoids.

### Base composition and codon usage

Similar to the mt genomes of other insects, the nucleotide composition of the *Euryomma* was A+T biased (A = 39.2%, T = 38.5%, G = 9.3%, C = 13.1%). *Delia* has a similar nucleotide composition (A = 38.9%, T = 38.3%, G = 9.6%, C = 13.2%). Average A+T content for the mt protein-coding genes across the six muscoid species sequenced to date is 75.8%. The A+T content for protein-coding genes on different strands (J-strand for *ND2*, *CO1*, *CO2*, *ATP8*, *ATP6*, *CO3*, *ND3*, *ND6*, and CYTB; N-strand for *ND1*, *ND4L*, *ND4*, and *ND5*) were calculated separately and are shown in Table 2. The AT-bias is stronger in N-strand PCGs than in J-strand PCGs, with J-strand being richer in C than G, and the N-strand showing an opposite skew for G and C. Strand bias in nucleotide composition was also assessed by analyzing the three codon positions separately, especially for the opposite GC-skew in the third codon position in two strands (J-strand: -0.49, N-strand: 0.67). AT-bias was stronger for RNA-encoding genes than in PCGs. Third codon positions, on which purifying selection against deleterious mutations is expected to be less severe, have a higher AT content than either the first and second codon position (A+T% at first / second / third codon position are 69.6% / 66.8% / 93.9%).

**Table 2:** Mitochondrial nucleotide composition in six muscoid flies.

Region		*Delia*	*Euryomma*.	*Haematobia*	*Musca*	*Stomoxys*	*Scathophaga*
**PCGs (J)**	A+T%	74.9	74.9	76.1	74.3	75.6	75.5
	G+C%	25.1	25.1	23.9	25.7	24.4	24.5
	AT-skew	-0.13	-0.13	-0.13	-0.12	-0.13	-0.13
	GC-skew	-0.08	-0.09	-0.04	-0.09	-0.07	-0.06
**1st condon position (J)**	A+T%	66.5	67.2	67.1	65.9	66.8	67
	G+C%	33.5	32.8	32.9	34.1	33.2	33
	AT-skew	-0.05	-0.05	-0.06	-0.05	-0.05	-0.06
	GC-skew	0.2	0.19	0.24	0.2	0.2	0.23
**2nd condon position (J)**	A+T%	65.4	65.3	65.5	65	65.7	65.3
	G+C%	34.6	34.7	34.5	35	34.3	34.7
	AT-skew	-0.38	-0.36	-0.36	-0.35	-0.36	-0.37
	GC-skew	-0.26	-0.27	-0.26	-0.27	-0.27	-0.26
**3rd condon position (J)**	A+T%	92.8	92.2	95.6	92.1	94.2	94.3
	G+C%	7.2	7.8	4.4	7.9	5.8	5.7
	AT-skew	0.003	-0.02	-0.02	-0.004	-0.04	-0.006
	GC-skew	-0.54	-0.47	-0.46	-0.5	-0.44	-0.53
**PCGs (N)**	A+T%	78.9	78.5	80.3	78.9	79.2	78.7
	G+C%	21.1	21.5	19.7	21.1	20.8	21.3
	AT-skew	-0.2	-0.2	-0.19	-0.19	-0.18	-0.2
	GC-skew	0.25	0.28	0.21	0.25	0.24	0.25
**1st condon position (N)**	A+T%	74.2	74.2	74.8	74	74.3	73.8
	G+C%	25.8	25.8	25.2	26	25.7	26.2
	AT-skew	-0.16	-0.14	-0.15	-0.18	-0.16	-0.15
	GC-skew	0.46	0.48	0.48	0.47	0.48	0.47
**2nd condon position (N)**	A+T%	68.6	68.6	68.8	68.8	69	68.2
	G+C%	31.4	31.4	31.2	31.2	31	31.8
	AT-skew	-0.43	-0.45	-0.42	-0.42	-0.43	-0.43
	GC-skew	-0.02	0	-0.04	-0.02	-0.03	-0.007
**3rd condon position (N)**	A+T%	93.9	92.7	97.1	93.9	94.2	94.1
	G+C%	6.1	7.3	2.9	6.1	5.8	5.9
	AT-skew	-0.07	-0.07	-0.05	-0.04	-0.007	-0.08
	GC-skew	0.74	0.77	0.61	0.68	0.59	0.62
**tRNA genes**	A+T%	77	77.5	71.6	77	77.4	76.4
	G+C%	23	22.5	28.4	23	22.6	23.6
	AT-skew	-0.02	0	-0.03	-0.01	-0.02	-0.01
	GC-skew	0.11	0.1	0.3	0.11	0.12	0.06
**lrRNA**	A+T%	82.4	82.8	82.7	81	82.6	82.4
	G+C%	17.6	17.3	17.3	19	17.4	17.6
	AT-skew	-0.03	-0.04	-0.01	-0.04	0	-0.01
	GC-skew	0.31	0.29	0.28	0.32	0.3	0.3
**srRNA**	A+T%	78.4	79.5	78.9	78.3	79.5	78.2
	G+C%	21.5	20.5	21.2	21.7	20.4	21.8
	AT-skew	-0.02	0	-0.04	-0.01	-0.03	0.01
	GC-skew	0.3	0.28	0.31	0.29	0.27	0.28
**Control region**	A+T%			89.4	89.7	87.5	89.5
	G+C%			10.5	10.3	12.5	10.5
	AT-skew			0.05	0.04	0.05	0.06
	GC-skew			-0.09	-0.17	0.01	-0.07
**Whole mitgenome**	A+T%			79.1	77.9	78.9	78.4
	G+C%			21	22.1	21.1	21.6
	AT-skew			0.01	0.01	-0.01	0.01
	GC-skew			-0.12	-0.16	-0.14	-0.14

Note: The A+T and G+C biases of protein-coding genes were calculated by AT-skew = [A-T]/[A+T] and GC-skew = [G-C]/[G+C], respectively.

Codon usage in the *Euryomma* and *Delia* mt genomes is shown in S5 and S6 Tables. Eleven of the 13 mt PCGs in both *Euryomma* and *Delia* used canonical ATN start codons. *ND2*, *ND3*, *ND5* and *ND6* used ATT (Ile), *ND4*, *ND4L*, *CO2*, *CO3*, *CytB* and *ATP6* used ATG (Met), and *ATP8* used ATC (Ile). *CO1* and *ND1* used non-canonical start codons, TCG (Ser) and TTG (Leu) respectively, which is normal amongst cyclorrhaphan mt genomes [54]. Similar patterns of start codon usage are found across the Muscoidea, with all genes using standard ATN start codons except for *CO1* and *ND1*. The use of a TCG start codons for *CO1* was consistent across the Muscoidea and is common in many other flies [36,55–58]. Using TTG as start codon for *ND1* has been considered as a common feature across Diptera [32], however, we found that *ND1* of all three Muscidae species consistently started with ATG.

The stop codon most commonly used in *Euryomma* is TAA (found in *ATP6*, *ATP8*, *CO1*, *CO3*, *CYTB*, *ND1*, *ND2*, *ND3*, *ND4*, *ND4L*, *ND6*), while the remaining two PCGs (*CO2*, *ND5*) utilise the partial stop codon T. *Delia* has the same pattern of stop codon use except for *ND4* which also utilizes a partial T stop codon in this species. The most commonly used stop codons in Muscoidea were TAA or TAG, while the stop codons in *CO2* and *ND5* were the partial T codon in all six species. A partial T stop codons for *ND4* was found in four of the six muscoid flies studied to date (*ND4* in *Musca* and *Euryomma* has a complete stop codon). Partial stop codons have been found in many insect mt genomes and are completed to a full TAA stop codons via post-transcriptional polyadenylation [59]. Diptera are no different and T or TA partial stop codons have been found in many other flies, including the genes *CO2*, *CYTB*, *ND1*, *ND2*, *ND3*, *ND4*, *ND5* and *CO1* [54].

### Transfer and ribosomal RNAs

All 22 standard tRNAs found in metazoan mt genomes were detected in both *Euryomma* and *Delia*; individual tRNAs ranged in size from 63 to 72 bp. Most tRNAs could be folded into the typical clover-leaf structure except for *tRNA* ^*Ser(AGN)*^ where the the DHU arm was absent (S1 and S2 Figs). The lack of a DHU arm in this gene has been commonly observed across metazoan mt genomes [35]. The anticodon and DHU stems had high levels of sequence conservation across the muscoid flies for all 22 tRNAs. Most variations within the tRNAs, including both nucleotide substitutions and indels, was found within the TΨC arms or on the DHU and variable loops. A total of 4 mismatched U–U base pairs were found in the *Euryomma* tRNA secondary structures, while 5 U–U mismatches were found in *Delia*. No other mismatch pairs were found in either mt genome.

**Fig 2:**
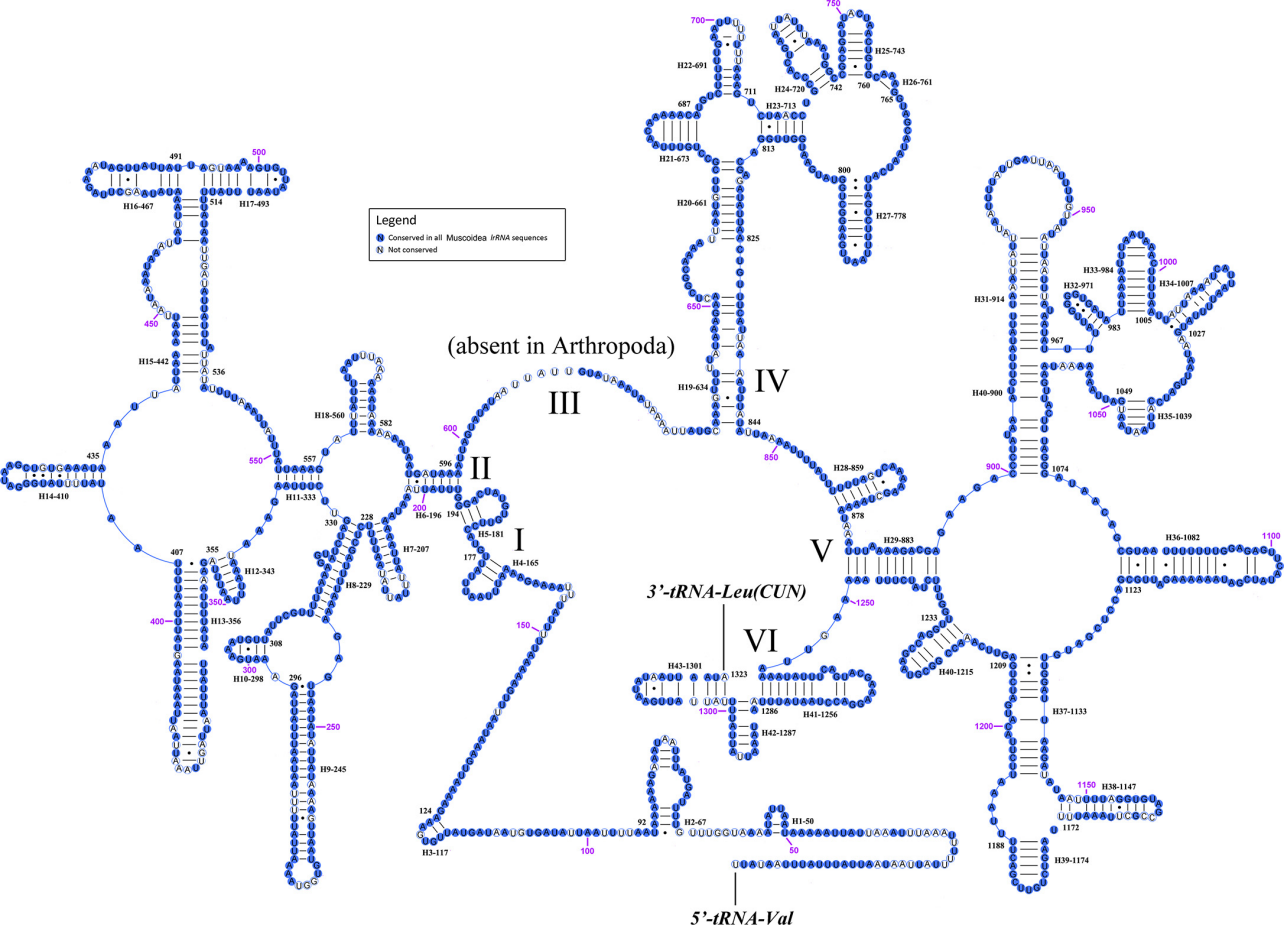
Predicted secondary structure of the *lrRNA* gene in muscoid flies. Gene sequence from *Delia* mt genome. Inferred Watson–Crick bonds are illustrated bylines, GU bonds by dots. Roman numerals denote the conserved domain structure. The darker circles indicate the conserved sites among all six muscoid species while the lighter circles indicate the unconserved sites.

**Fig 3:**
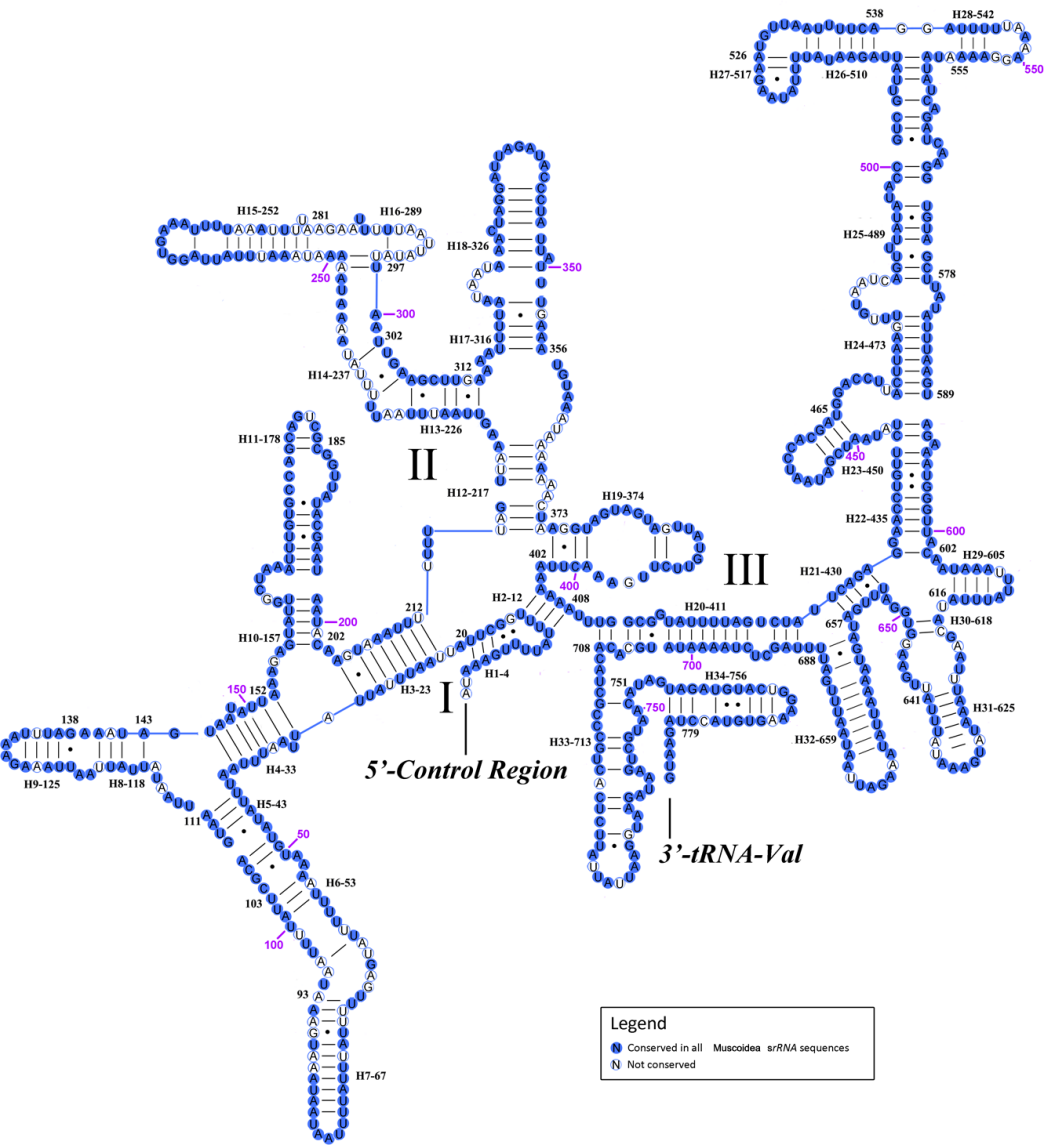
Predicted secondary structure of the *srRNA* gene in muscoid flies. Gene sequence from *Delia* mt genome. Inferred Watson–Crick bonds are illustrated bylines, GU bonds by dots. Roman numerals denote the conserved domain structure. The darker circles indicate the conserved sites among all six muscoid species while the lighter circles indicate the unconserved sites.

We inferred secondary structures of *lrRNA* and *srRNA* of muscoid flies using the published rRNA secondary structures of *Drosophila yakuba* (Diptera) [20], *Apis mellifera* (Hymenoptera) [26], and *Libelloides macaronius* (Neuroptera) [27] as reference [54]. *lrRNA* has 49 helices in five structural domains (I-II, IV-VI, domain III is absent as in other Arthropoda), similar to other arthropods [60] (Fig 2). The secondary structures of *srRNA* in Muscoidea include three domains and 33 helices, similar to other Diptera [55] (Fig 3). Due to the high level of sequence conservation within the rRNA genes of muscoids, such variability as was present was mapped onto the secondary structure inferred for *Delia* (sites conserved in all 6 species marked in dark blue, variable sites uncoloured; Figs 2 and 3).

### Phylogeny

**Fig 4:**
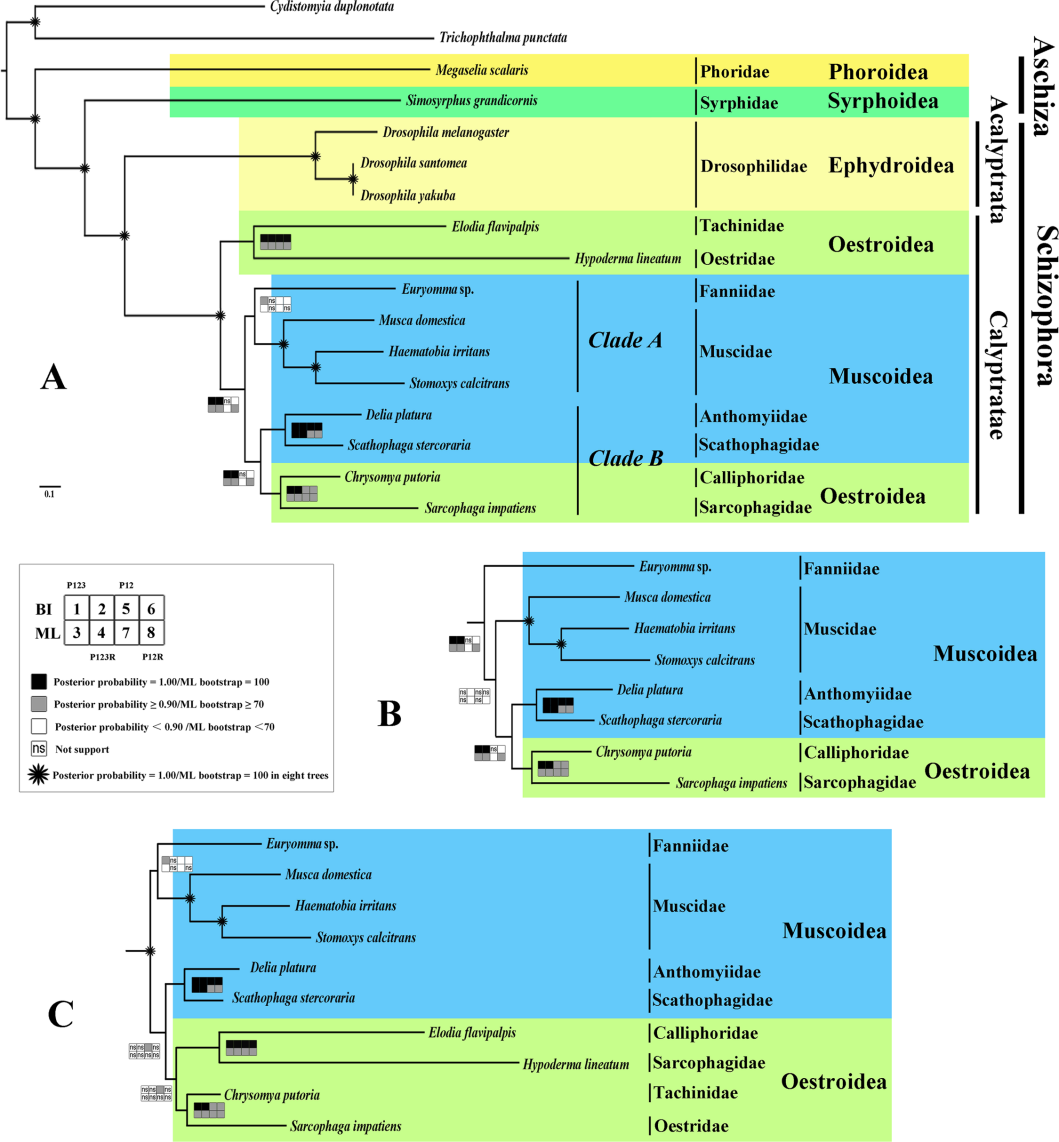
Phylogenetic tree based on mt genome data. Cladogram of relationships resulting from Bayesian analyses with datasets PCG123 & PCG12RNA and ML analyses with datasets PCG12, with *Cydistomyia duplonotata* (Nemestrinidae) and *Trichophthalma punctata* (Tabanidae) as outgroups. Squares at the nodes are Bayesian posterior probabilities for1, 2, 5 and 6, ML bootstrap values for 3, 4, 7 and 8. Dataset of PCG123, 1 and 3, PCG123RNA, 2 and 4, PCG12, 5 and 7, PCG12RNA, 6 and 8. Black indicates posterior probabilities = 1.00 or ML bootstrap = 100, gray indicates posterior probabilities≥ 0.90 or ML bootstrap≥ 70, white indicates posterior probabilities< 0.90 or ML bootstrap< 70, ‘ns’ = not support, * indicates posterior probabilities = 1.00 or ML bootstrap = 100 in eight trees. A. The Bayesian tree of datasets PCG123 and PCG12RNA as well as ML tree of datasets PCG12 and PCG123. B. Part of the Bayesian tree of dataset PCG123RNA as well as ML tree of datasets PCG123RNA and PCG12RNA. C. Part of the Bayesian tree of dataset PCG12.

Phylogenetic trees were inferred using two approaches (BI and ML) for four datasets that differ by including or excluding third codon positions or RNA genes or both (PCG123, PCG123RNA, PCG12, and PCG12RNA) (Fig 4). All three topologies inferred from four datasets were exhibited in Fig 4. The topology of BI-P123, BI-12R, ML-P12, as well as ML-P123 were Fig 4A, the topology of BI-P123R, ML-P12R,and ML-P123R were Fig 4B, the Fig 4C represented BI-P12 only. The monophyly of the Schizophora and Calyptratae were consistently supported (posterior probability = 1.00, ML bootstrap = 100% in all four dataset). The Aschiza was paraphyletic, as is commonly accepted [5, 53, 61–66]. Phoridae was sister to the remaining Cyclorrhapha (PP 1.00, ML 100% in all four datasets). These results are same as recent wide-scale molecular [5] and morphological [7] studies regarding the branching order within the Aschiza.

Phylogenetic relationships among the Muscoidea and Oestroidea remains controversial [67]. Michelsen et al. suggested that Anthomyiidae and Muscidae were sister groups, whereas it was also been suggested that the sister-group relationship is between Muscidae and Fanniidae [6]. The relationship Fanniidae + (Scathophagidae + (Muscidae + Anthomyiidae)) was proposed by Pont (1998) on the basis of 25 characters, including adult and larval morphology, plus larval food characters. Based on four mitochondrial and four nuclear genes sequenced from 127 exemplar species, Kutty et al. (2008) found that Muscoidea was paraphyletic with a monophyletic Oestroidea nested within Muscoidea as sister to a clade composed of Anthomyiidae (which was grossly paraphyletic) and Scathophagidae, although none of the interfamilial nodes were significantly supported. These relationships were subsequently supported by Kutty et al. (2010) and Wiegmann et al. (2011), which both found significant nodal support for Oestroidea + (Anthomyiidae+Scathophagidae), but support for other interfamilial nodes was not significant. The robust inference of relationships within the Muscoidea and Oestroidea still needs further investigation, so we tested the phylogenetic position of each of the four families in the Muscoidea.

Only one of the eight analyses supported the monophyly of the Oestroidea, the Bayesian analysis of dataset PCG12 (PP = 0.993) (Fig 4C), the other seven analyses supported a paraphyletic Oestroidea, with Muscoidea nested within Oestroidea, as sister to Calliphoridae+Sarcophagidae. This result is different from most previous studies which found Oestroidea to be monophyletic, and Muscoidea as a paraphyletic grade at the base of Oestroidea [1, 4, 5, 7]. Two previous studies have used complete mt genomic data [32, 36], however these studies have included only a single species from the Muscoidea, and each study found that the monophyly of the Oestroidea was sensitive to analytical method with some analyses grouping Muscidae with Calliphoridae [36] or with the clade (Sarcophagidae+Calliphoidae) [32].

Our results provide further evidence in support of a paraphyletic ‘Muscoidea’, consistent with previous studies [4]. The clade Fanniidae + Muscidae (clade A, Fig 4A) was inferred as a sister group to ((Anthomyiidae + Scathophagidae) + (Calliphoridae + Sarcophagidae)) (clade B) in 4 of the 8 analyses, albeit with poor nodal support (insignificant in 3 analyses; >0.90PP, >70% BS in the fourth analysis). In 3 of the 8 analyses (BI-PCG123R, ML-PCG123R, ML-PCG12R), Fanniidae was placed as sister to a clade composed of the remaining muscoids, including the clade Calliphoridae+Sarcophagidae. In the final analysis (BI-PCG12), the muscoids were paraaphyletic with respect to a monophyletic Oestroidea, as discussed above: (Fanniidae + Muscidae) + ((Anthomyiidae + Scathophagidae) + Oestroidea). In all analyses Scathophagidae is sister to Anthomyiidae and relative derived within the muscoidea (i.e. sister to part or all of the Oestroidea). These results contradict previous conclusions based on morphological characters that Scathophagidae retained a more plesiomorphic conditions than any other group [68].

The different topologies in our study may be due to the compositional bias of mt genome data, particularly at third codon positions as has been proposed for other insect mt genome phylogenies [28, 32, 54, 69]. In this study, however, removal of third codon positions did not stabilize the topology as all three recovered topologies were found in the subset of 4 analyses that omitted third codon positions. Further studies with more sampling species are crucial for better understanding relationships within the Cyclorrhapha.

### Estimation of Divergence Times

**Fig 5:**
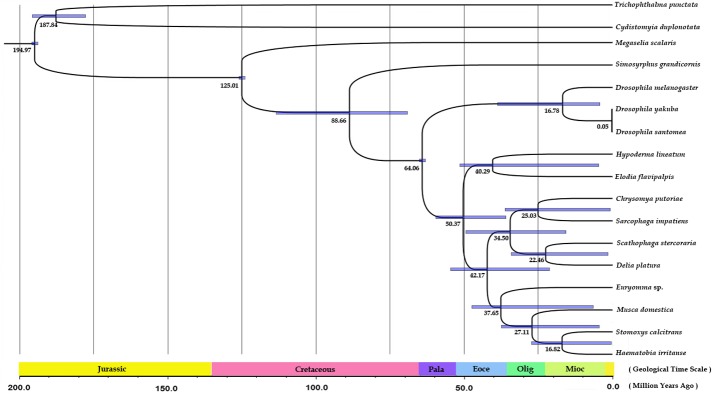
Evolutionary timescale for Calyptratae inferred from a mitochondrial PCG123 dataset. Numbers at nodes indicate mean estimated divergence times (in mya) and node bars indicate 95% credibility intervals. In the geological time scale: Pala indicates Palaeocene; Eoce indicates Eocene; Oligo indicate Oligocene; Mioc indicated Miocene.

A Bayesian, relaxed-clock dating method was used to estimate divergence times within the Calyptratae, the maximum clade credibility tree with median node heights and the 95% high posterior density (HPD) interval on each divergence (Fig 5). Of the 4 alignment datasets used to estimate muscoid phylogeny, PCG123 was used to estimate divergence times as it had the highest node support in initial phylogenetic assessment and the topology of this dataset was the most representative one (the main tree) among all of datasets. Molecular dating based on the PCG123 dataset suggested that the most recent ancestor of Oestroidea and Muscoidea existed in the early Eocene (~50 mya) (95% HPD 42–58 mya). This result is consistent with the evidence from a number of amber specimens of Acalyptrata and Calyptratae [70]. The earliest divergence within the Calyptratae, the split between clade A and clade B, was estimated to date to the middle Eocene (~42 mya) (95% HPD 34–52 mya). Subsequently, the divergence of Muscidae and Fanniidae is estimated to have occurred in the late Eocene (~38 mya) (95% HPD 28–48 mya), while the Scathophagidae and Anthomyiidae diverge in the early Miocene (~22 mya) (95% HPD 9–35 mya).

Our estimation of interfamilial divergences of Muscoidea shows broad confidence intervals, suggesting possible limitations in the use of mt genomic data for divergence time estimation. First, broad confidence intervals may reflect difficulties in modeling the inherently heterogeneous patterns of mutation of various PCGs in the mt genome [71–72]. Alternatively, when using all the three sites of PCGs to estimate the divergence time, the saturated nucleotide sites may overestimate the branching times [73].

In general, the phylogeny inferred from mt genomes indicated that both the superfamily Muscoidea and Oestroidea were polyphyletic, and that interfamily relationships among Muscoidea and Oestroidea are still unresolved. It is worth noting that mt genes are physically linked, and they act as a single locus, so they may alternatively simply illustrate the evolution of mt genomes within this clade, the analysis of additional taxa and nuclear gene data would be helpful for better understanding the relationships of Calyptratae.

## Supporting Information

S1 Fig Putative secondary strutrues of tRNAs found in the mt genome of *Euryomma* sp. (TIF)

S2 Fig Putative secondary strutrues of tRNAs found in the mt genome of *Delia platura*. (TIF)

S1 Table Primers used in this study. (DOCX)

S2 Table The best partitioning scheme selected by PartitionFinder for different dataset. (DOCX)

S3 Table Organization of the mt genome of *Euryomma* sp. (DOCX)

S4 Table Organization of the mt genome of *Delia platura*. (DOCX)

S5 Table Codon usage of the *Euryomma* sp. mt genome. (DOCX)

S6 Table Codon usage of the *Delia platura* mt genome. (DOCX)
