# Supplementary figures and images for: The Phylogeny and Evolutionary Timescale of Muscoidea (Diptera: Brachycera: Calyptratae) Inferred from Mitochondrial Genomes
Source: PLoS One. 2015 Jul 30;10(7):e0134170. doi: 10.1371/journal.pone.0134170 (PMC4520480; doi:10.1371/journal.pone.0134170)

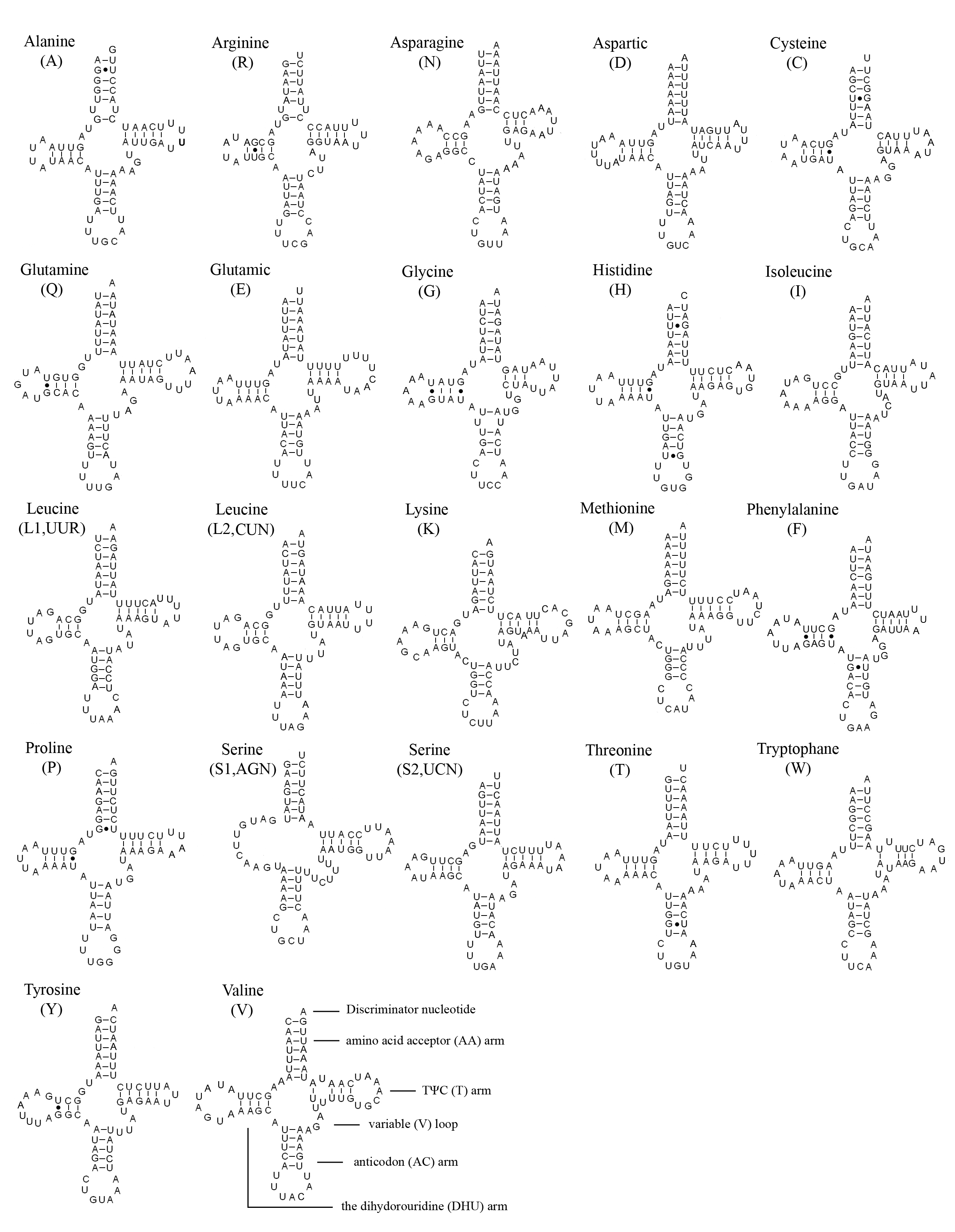

Supplement: S1 Fig — (TIF) [file pone.0134170.s001.tif]

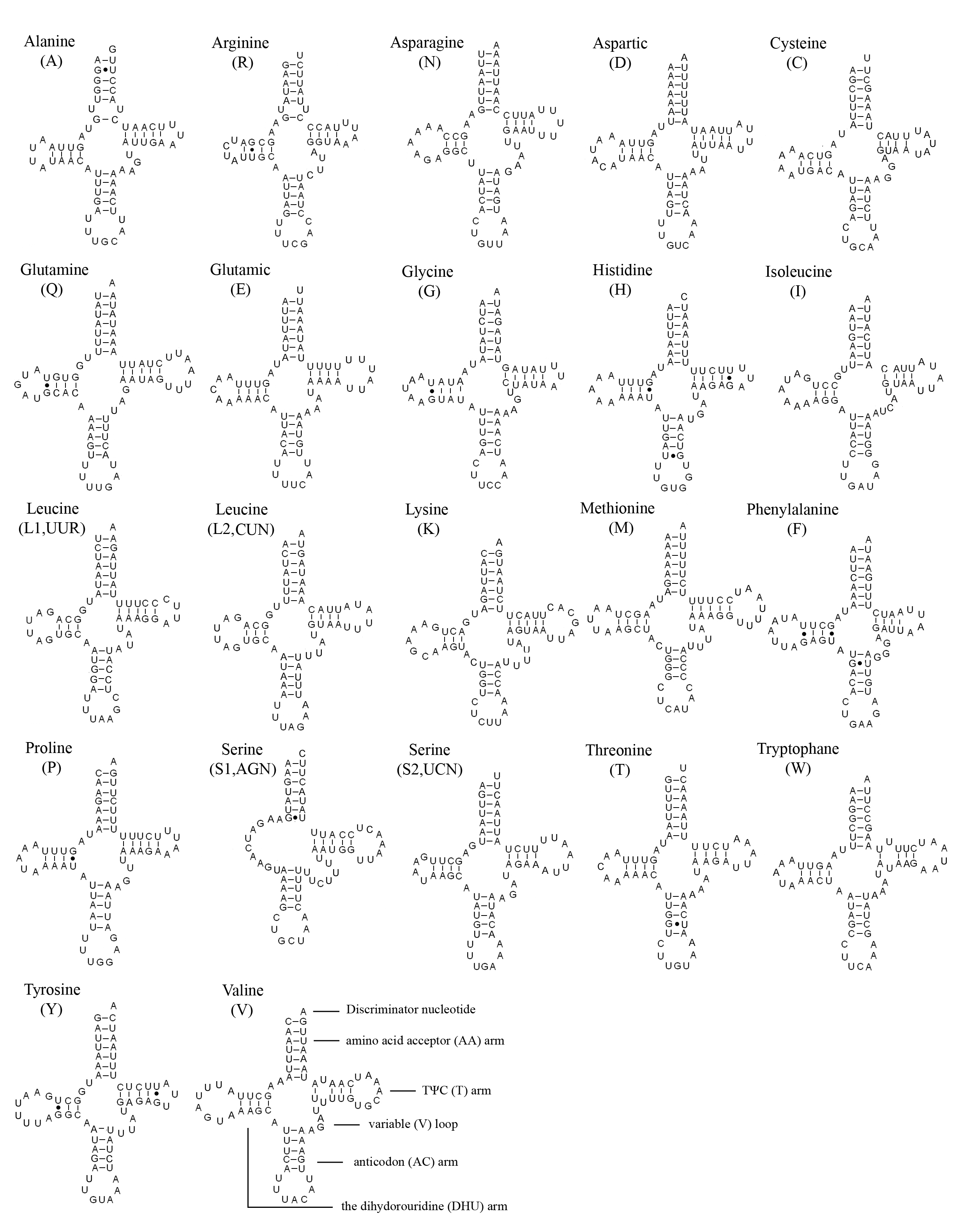

Supplement: S2 Fig — (TIF) [file pone.0134170.s002.tif]
