# Supplementary material for: The Phylogeny and Evolutionary Timescale of Muscoidea (Diptera: Brachycera: Calyptratae) Inferred from Mitochondrial Genomes
Source: PLoS One. 2015 Jul 30;10(7):e0134170. doi: 10.1371/journal.pone.0134170 (PMC4520480; doi:10.1371/journal.pone.0134170)
Supplement: S2 Table — (DOCX) [file pone.0134170.s004.docx]

**Table S2. The best partitioning scheme selected by PartitionFinder for different dataset.**

| **Dataset** | **Subset Partitions** | **Best Model** |
| --- | --- | --- |
| PCG123RNA:  7 partitions (BI) | P1: (ATP6_pos1, CO1_pos1, CO2_pos1, CO3_pos1, CytB_pos1, tRNAs) | GTR+I+G |
|  | P2: (ATP6_pos2, ATP8_pos2, CO1_pos2, CO2_pos2, CO3_pos2, CytB_pos2, ND1_pos2, ND2_pos2, ND3_pos2, ND4L_pos2, ND4_pos2, ND5_pos2, ND6_pos2) | GTR+I+G |
|  | P3: (ATP6_pos3, CO1_pos3, CO2_pos3, CO3_pos3, CytB_pos3, ND2_pos3, ND3_pos3, ND6_pos3) | GTR+I+G |
|  | P4: (ATP8_pos1, ATP8_pos3, ND2_pos1, ND3_pos1, ND6_pos1) | GTR+I+G |
|  | P5: (12S_NT, 16S_NT, ND1_pos1, ND4L_pos1, ND4_pos1, ND5_pos1) | GTR+I+G |
|  | P6: (ND1_pos3, ND4L_pos3, ND5_pos3) | HKY+I+G |
|  | P7: (ND4_pos3) | HKY+I+G |
| PCG123RNA:  5 partitions (ML) | P1: (ATP6_pos1, CO1_pos1, CO2_pos1, CO3_pos1, CytB_pos1, tRNA) | GTR+I+G |
|  | P2: (ATP6_pos2, ATP8_pos2, CO1_pos2, CO2_pos2, CO3_pos2, CytB_pos2, ND1_pos2, ND2_pos2, ND3_pos2, ND4L_pos2, ND4_pos2, ND5_pos2, ND6_pos2) | GTR+I+G |
|  | P3: (ATP6_pos3, ATP8_pos3, CO1_pos3, CO2_pos3, CO3_pos3, CytB_pos3, ND2_pos3, ND3_pos3, ND6_pos3) | GTR+I+G |
|  | P4: (12S_NT, 16S_NT, ATP8_pos1, ND1_pos1, ND2_pos1, ND3_pos1, ND4L_pos1, ND4_pos1, ND5_pos1, ND6_pos1) | GTR+I+G |
|  | P5: (ND1_pos3, ND4L_pos3, ND4_pos3, ND5_pos3) | GTR+I+G |
| PCG123:  6 partitions (BI) | P1: (ATP6_pos1, CO1_pos1, CO2_pos1, CO3_pos1, CytB_pos1) | GTR+I+G |
|  | P2: (ATP6_pos2, CO1_pos2, CO2_pos2, CO3_pos2, CytB_pos2, ND1_pos2, ND2_pos2, ND3_pos2, ND4L_pos2, ND4_pos2, ND5_pos2, ND6_pos2) | GTR+I+G |
|  | P3: (ATP6_pos3, ATP8_pos3, CO1_pos3, CO2_pos3, CO3_pos3, CytB_pos3, ND2_pos3, ND3_pos3, ND6_pos3) | GTR+I+G |
|  | P4: (ATP8_pos1, ATP8_pos2, ND1_pos1, ND2_pos1, ND3_pos1, ND4L_pos1, ND4_pos1, ND5_pos1, ND6_pos1) | GTR+I+G |
|  | P5: (ND1_pos3, ND4L_pos3, ND5_pos3) | HKY+I+G |
|  | P6: (ND4_pos3) | HKY+I+G |
| PCG123:  6 partitions (ML) | P1: (ATP6_pos1, CO1_pos1, CO2_pos1, CO3_pos1, CytB_pos1) | GTR+I+G |
|  | P2: (ATP6_pos2, ATP8_pos2, CO1_pos2, CO2_pos2, CO3_pos2, CytB_pos2, ND1_pos2, ND2_pos2, ND3_pos2, ND4L_pos2, ND4_pos2, ND5_pos2, ND6_pos2) | GTR+I+G |
|  | P3: (ATP6_pos3, ATP8_pos3, CO1_pos3, CO2_pos3, CO3_pos3, CytB_pos3, ND2_pos3, ND3_pos3, ND6_pos3) | GTR+I+G |
|  | P4: (ATP8_pos1, ND1_pos1, ND2_pos1, ND3_pos1, ND4L_pos1, ND4_pos1, ND5_pos1, ND6_pos1) | GTR+I+G |
|  | P5: (ND1_pos3, ND4L_pos3, ND5_pos3) | GTR+I+G |
|  | P6: (ND4_pos3) | GTR+I+G |
| PCG12RNA  4 partitions (BI) | P1: (ATP6_pos1, CO1_pos1, CO2_pos1, CO3_pos1, CytB_pos1) | GTR+I+G |
|  | P2: (ATP6_pos2, CO1_pos2, CO2_pos2, CO3_pos2, CytB_pos2, ND1_pos2, ND2_pos2, ND3_pos2, ND4L_pos2, ND4_pos2, ND5_pos2, ND6_pos2) | GTR+I+G |
|  | P3: (ATP8_pos1, ATP8_pos2, ND2_pos1, ND3_pos1, ND6_pos1) | GTR+I+G |
|  | P4: (ND1_pos1, ND4L_pos1, ND4_pos1, ND5_pos1, 12S, 16S, tRNAs) | GTR+I+G |
| PCG12RNA:  4 partitions (ML) | P1: (ATP6_pos1, CO1_pos1, CO2_pos1, CO3_pos1, CytB_pos1) | GTR+I+G |
|  | P2: (ATP6_pos2, ATP8_pos2, CO1_pos2, CO2_pos2, CO3_pos2, CytB_pos2, ND1_pos2, ND2_pos2, ND3_pos2, ND4_pos2, ND5_pos2, ND6_pos2) | GTR+I+G |
|  | P3: (ATP8_pos1, ND2_pos1, ND3_pos1, ND6_pos1) | GTR+I+G |
|  | P4: (ND1_pos1, ND4L_pos1, ND4L_pos2, ND4_pos1, ND5_pos1, 12S, 16S, tRNAs) | GTR+I+G |
| PCG12:  3 partitions (BI) | P1: (ATP6_pos1, CO1_pos1, CO2_pos1, CO3_pos1, CytB_pos1) | GTR+I+G |
|  | P2: (ATP6_pos2, ATP8_pos2, CO1_pos2, CO2_pos2, CO3_pos2, CytB_pos2, ND1_pos2, ND2_pos2, ND3_pos2, ND4L_pos2, ND4_pos2, ND5_pos2, ND6_pos2) | GTR+I+G |
|  | P3: (ATP8_pos1, ND1_pos1, ND2_pos1, ND3_pos1, ND4L_pos1, ND4_pos1, ND5_pos1, ND6_pos1) | GTR+I+G |
| PCG12:  3 partitions (ML) | P1: (ATP6_pos1, CO1_pos1, CO2_pos1, CO3_pos1, CytB_pos1) | GTR+I+G |
|  | P2: (ATP6_pos2, ATP8_pos2, CO1_pos2, CO2_pos2, CO3_pos2, CytB_pos2, ND1_pos2, ND2_pos2, ND3_pos2, ND4_pos2, ND5_pos2, ND6_pos2) | GTR+I+G |
|  | P3: (ATP8_pos1, ND1_pos1, ND2_pos1, ND3_pos1, ND4L_pos1, ND4L_pos2, ND4_pos1, ND5_pos1, ND6_pos1) | GTR+I+G |
