# Supplementary material for: The Phylogeny and Evolutionary Timescale of Muscoidea (Diptera: Brachycera: Calyptratae) Inferred from Mitochondrial Genomes
Source: PLoS One. 2015 Jul 30;10(7):e0134170. doi: 10.1371/journal.pone.0134170 (PMC4520480; doi:10.1371/journal.pone.0134170)
Supplement: S3 Table — (DOCX) [file pone.0134170.s005.docx]

**Table S3. Organization of the mt genome of** ***Euryomma* sp.**

| Gene | Direction | Location | Size (bp) | IGN*1215 | Anticodon | Codon | | AT% |
| --- | --- | --- | --- | --- | --- | --- | --- | --- |
|  |  |  |  |  |  | Start | Stop |  |
| tRNA^Ile^ | F | 1–66 | 66 |  | GAT |  |  | 74.2 |
| tRNA^Gln^ | R | 67–135 | 69 | 0 | TTG |  |  | 81.1 |
| tRNA^Met^ | F | 140–208 | 69 | 4 | CAT |  |  | 71.0 |
| *ND2* | F | 209–1225 | 1017 | 0 |  | ATT | TAA | 80.1 |
| tRNA^Trp^ | F | 1224–1291 | 68 | -2 | TCA |  |  | 76.5 |
| tRNA^Cys^ | R | 1298–1361 | 64 | 6 | GCA |  |  | 73.4 |
| tRNA^Tyr^ | R | 1370–1435 | 66 | 8 | GTA |  |  | 74.2 |
| *CO1* | F | 1434–2972 | 1539 | -2 |  | TCG | TAA | 70.4 |
| tRNA^Leu(UUR)^ | F | 2968–3033 | 66 | -5 | TAA |  |  | 74.2 |
| *CO2* | F | 3038–3725 | 688 | 4 |  | ATG | T | 75.2 |
| tRNA^Lys^ | F | 3726–3796 | 71 | 0 | CTT |  |  | 69.0 |
| tRNA^Asp^ | F | 3799–3865 | 67 | 2 | GTC |  |  | 89.6 |
| *ATP8* | F | 3866–4030 | 165 | 0 |  | ATC | TAA | 81.8 |
| *ATP6* | F | 4024–4701 | 678 | -7 |  | ATG | TAA | 74.5 |
| *CO3* | F | 4701–5489 | 789 | -1 |  | ATG | TAA | 69.9 |
| tRNA^Gly^ | F | 5500–5564 | 65 | 10 | TCC |  |  | 83.1 |
| *ND3* | F | 5565–5918 | 354 | 0 |  | ATT | TAA | 78.0 |
| tRNA^Ala^ | F | 5923–5986 | 64 | 4 | TGC |  |  | 73.4 |
| tRNA^Arg^ | F | 5986–6048 | 63 | -1 | TCG |  |  | 68.3 |
| tRNA^Asn^ | F | 6054–6118 | 65 | 5 | GTT |  |  | 75.4 |
| tRNA^Ser(AGN)^ | F | 6119–6186 | 68 | 0 | GCT |  |  | 72.1 |
| tRNA^Glu^ | F | 6214–6279 | 66 | 27 | TTC |  |  | 90.9 |
| tRNA^Phe^ | R | 6298–6363 | 66 | 18 | GAA |  |  | 74.2 |
| *ND5* | R | 6364–8083 | 1720 | 0 |  | ATT | T | 78.6 |
| tRNA^His^ | R | 8099–8163 | 65 | 15 | GTG |  |  | 81.6 |
| *ND4* | R | 8163–9503 | 1341 | -1 |  | ATG | TAA | 78.6 |
| *ND4L* | R | 9503–9793 | 291 | -1 |  | ATG | TAA | 81.8 |
| tRNA^Thr^ | F | 9796–9860 | 65 | 2 | TGT |  |  | 81.6 |
| tRNA^Pro^ | R | 9861–9926 | 66 | 0 | TGG |  |  | 81.8 |
| *ND6* | F | 9929–10453 | 525 | 2 |  | ATT | TAA | 83.6 |
| *CYTB* | F | 10453–11589 | 1137 | -1 |  | ATG | TAA | 73.8 |
| tRNA^Ser(UCN)^ | F | 11589–11654 | 66 | -1 | TGA |  |  | 78.8 |
| *ND1* | R | 11671–12618 | 948 | 16 |  | TTG | TAA | 77.3 |
| tRNA^Leu(CUN)^ | R | 12620–12684 | 65 | 1 | TAG |  |  | 81.6 |
| lrRNA | R | 12685–14017 | 1333 | 0 |  |  |  | 82.8 |
| tRNA^Val^ | R | 14018–14089 | 72 | 0 | TAC |  |  | 79.2 |
| srRNA | R | 14090–14877 | 788 | 0 |  |  |  | 79.5 |
| Control region (part) | - | 14878–15315 |  | 438+ |  |  |  |  |

Note: * IGN: Intergenic nucleotide, minus indicates overlapping between genes. tRNAX: where X is the abbreviation of the corresponding amino acid.
