# Supplementary material for: The Phylogeny and Evolutionary Timescale of Muscoidea (Diptera: Brachycera: Calyptratae) Inferred from Mitochondrial Genomes
Source: PLoS One. 2015 Jul 30;10(7):e0134170. doi: 10.1371/journal.pone.0134170 (PMC4520480; doi:10.1371/journal.pone.0134170)
Supplement: S4 Table — (DOCX) [file pone.0134170.s006.docx]

**Table S4. Organization of the mt genome of *Delia platura***

| Gene | Direction | Location | Size (bp) | IGN* | Anticodon | Codon | | AT% |
| --- | --- | --- | --- | --- | --- | --- | --- | --- |
|  |  |  |  |  |  | Start | Stop |  |
| tRNA^Ile^ | F | 1–66 | 66 |  | GAT |  |  | 74.2 |
| tRNA^Gln^ | R | 64–132 | 69 | -3 | TTG |  |  | 81.1 |
| tRNA^Met^ | F | 132–200 | 69 | -1 | CAT |  |  | 69.6 |
| *ND2* | F | 201–1217 | 1017 | 0 |  | ATT | TAA | 80.2 |
| tRNA^Trp^ | F | 1217–1284 | 68 | -1 | TCA |  |  | 75.0 |
| tRNA^Cys^ | R | 1277–1340 | 64 | -8 | GCA |  |  | 73.5 |
| tRNA^Tyr^ | R | 1351–1416 | 66 | 10 | GTA |  |  | 74.3 |
| *CO1* | F | 1428–2966 | 1539 | 11 |  | TCG | TAA | 69.8 |
| tRNA^Leu(UUR)^ | F | 2962–3027 | 66 | -5 | TAA |  |  | 75.8 |
| *CO2* | F | 3037–3724 | 688 | 9 |  | ATG | T | 74.3 |
| tRNA^Lys^ | F | 3725–3795 | 71 | 0 | CTT |  |  | 67.6 |
| tRNA^Asp^ | F | 3798–3865 | 68 | 2 | GTC |  |  | 88.2 |
| *ATP8* | F | 3866–4030 | 165 | 0 |  | ATC | TAA | 83.0 |
| *ATP6* | F | 4024–4701 | 678 | -7 |  | ATG | TAA | 74.2 |
| *CO3* | F | 4701–5489 | 789 | -1 |  | ATG | TAA | 71.1 |
| tRNA^Gly^ | F | 5496–5560 | 65 | 6 | TCC |  |  | 80.0 |
| *ND3* | F | 5561–5914 | 354 | 0 |  | ATT | TAA | 78.2 |
| tRNA^Ala^ | F | 5922–5986 | 65 | 7 | TGC |  |  | 73.9 |
| tRNA^Arg^ | F | 5986–6049 | 64 | -1 | TCG |  |  | 70.3 |
| tRNA^Asn^ | F | 6062–6127 | 66 | 12 | GTT |  |  | 72.8 |
| tRNA^Ser(AGN)^ | F | 6128–6195 | 68 | 0 | GCT |  |  | 73.5 |
| tRNA^Glu^ | F | 6196–6263 | 68 | 0 | TTC |  |  | 91.2 |
| tRNA^Phe^ | R | 6282–6348 | 67 | 18 | GAA |  |  | 76.1 |
| *ND5* | R | 6348–8068 | 1720 | 0 |  | ATT | T | 78.8 |
| tRNA^His^ | R | 8084–8150 | 67 | 15 | GTG |  |  | 77.6 |
| *ND4* | R | 8149–9489 | 1341 | -2 |  | ATG | T | 78.8 |
| *ND4L* | R | 9489–9779 | 291 | -1 |  | ATG | TAA | 82.1 |
| tRNA^Thr^ | F | 9782–9845 | 64 | 2 | TGT |  |  | 82.8 |
| tRNA^Pro^ | R | 9846–9910 | 65 | 0 | TGG |  |  | 77.3 |
| *ND6* | F | 9914–10438 | 525 | 2 |  | ATT | TAA | 84.0 |
| *CYTB* | F | 10438–11574 | 1137 | -1 |  | ATG | TAA | 74.4 |
| tRNA^Ser(UCN)^ | F | 11577–11644 | 68 | 2 | TGA |  |  | 79.4 |
| *ND1* | R | 11661–12608 | 948 | 16 |  | TTG | TAA | 78.5 |
| tRNA^Leu(CUN)^ | R | 12610–12674 | 65 | 1 | TAG |  |  | 81.5 |
| lrRNA | R | 12680–14002 | 1323 | 5 |  |  |  | 82.4 |
| tRNA^Val^ | R | 14003–14074 | 72 | 0 | TAC |  |  | 79.2 |
| srRNA | R | 14075–14858 | 784 | 0 |  |  |  | 78.4 |

Note: * IGN: Intergenic nucleotide, minus indicates overlapping between genes. tRNAX: where X is the abbreviation of the corresponding amino acid.
