# Supplementary material for: The Phylogeny and Evolutionary Timescale of Muscoidea (Diptera: Brachycera: Calyptratae) Inferred from Mitochondrial Genomes
Source: PLoS One. 2015 Jul 30;10(7):e0134170. doi: 10.1371/journal.pone.0134170 (PMC4520480; doi:10.1371/journal.pone.0134170)
Supplement: S5 Table — (DOCX) [file pone.0134170.s007.docx]

**Table S5.** **Codon usage of the *Euryomma*. sp. mt genome.**

| Amino acid | Codon | N | RSCU | N+ | RSCU | N- | RSCU |
| --- | --- | --- | --- | --- | --- | --- | --- |
| Phe (F) | **UUU** | **313** | **1.87** | **182** | **1.8** | **131** | **1.97** |
|  | UUC | 22 | 0.13 | 20 | 0.2 | 2 | 0.03 |
| Leu^UUR^ (L) | **UUA** | **515** | **5.1** | **282** | **4.92** | **233** | **5.34** |
|  | UUG | 28 | 0.28 | 7 | 0.12 | 21 | 0.48 |
| Leu^CUN^ (L) | **CUU** | **33** | **0.33** | **26** | **0.45** | **7** | **0.16** |
|  | CUC | 1 | 0.01 | 1 | 0.02 | 0 | 0 |
|  | CUA | 28 | 0.28 | 27 | 0.47 | 1 | 0.02 |
|  | CUG | 1 | 0.01 | 1 | 0.02 | 0 | 0 |
| Ile (I) | **AUU** | **353** | **1.9** | **225** | **1.86** | **128** | **1.98** |
|  | AUC | 18 | 0.1 | 17 | 0.14 | 1 | 0.02 |
| Met (M) | **AUA** | **208** | **1.79** | **112** | **1.84** | **96** | **1.73** |
|  | AUG | 25 | 0.21 | 10 | 0.16 | 15 | 0.27 |
| Val (V) | **GUU** | **87** | **1.86** | **42** | **1.54** | **45** | **2.31** |
|  | GUC | 6 | 0.13 | 5 | 0.18 | 1 | 0.05 |
|  | GUA | 86 | 1.84 | 59 | 2.17 | 27 | 1.38 |
|  | GUG | 8 | 0.17 | 3 | 0.11 | 5 | 0.26 |
| Ser^UCN^ (S) | **UCU** | **124** | **2.93** | **62** | **2.64** | **62** | **3.28** |
|  | UCC | 6 | 0.14 | 6 | 0.26 | 0 | 0 |
|  | UCA | 101 | 2.38 | 78 | 3.32 | 23 | 1.22 |
|  | UCG | 4 | 0.09 | 2 | 0.09 | 2 | 0.11 |
| Pro (P) | **CCU** | **83** | **2.46** | **60** | **2.33** | **23** | **2.88** |
|  | CCC | 3 | 0.09 | 3 | 0.12 | 0 | 0 |
|  | CCA | 47 | 1.39 | 39 | 1.51 | 8 | 1 |
|  | CCG | 2 | 0.06 | 1 | 0.04 | 1 | 0.13 |
| Thr (T) | **ACU** | **103** | **2.18** | **72** | **2.03** | **31** | **2.64** |
|  | ACC | 12 | 0.25 | 11 | 0.31 | 1 | 0.09 |
|  | ACA | 71 | 1.5 | 58 | 1.63 | 13 | 1.11 |
|  | ACG | 3 | 0.06 | 1 | 0.03 | 2 | 0.17 |
| Ala (A) | **GCU** | **111** | **2.55** | **69** | **2.4** | **42** | **2.85** |
|  | GCC | 15 | 0.34 | 13 | 0.45 | 2 | 0.14 |
|  | GCA | 43 | 0.99 | 31 | 1.08 | 12 | 0.81 |
|  | GCG | 5 | 0.11 | 2 | 0.07 | 3 | 0.2 |
| Tyr (Y) | **UAU** | **152** | **1.84** | **78** | **1.75** | **74** | **1.95** |
|  | UAC | 13 | 0.16 | 11 | 0.25 | 2 | 0.05 |
| Stop (*) | **UAA** | **11** | **2** | **8** | **2** | **3** | **2** |
|  | UAG | 0 | 0 | 0 | 0 | 0 | 0 |
| His (H) | **CAU** | **65** | **1.76** | **52** | **1.7** | **13** | **2** |
|  | CAC | 9 | 0.24 | 9 | 0.3 | 0 | 0 |
| Gln (Q) | **CAA** | **68** | **1.92** | **47** | **2** | **21** | **1.75** |
|  | CAG | 3 | 0.08 | 0 | 0 | 3 | 0.25 |
| Asn (N) | **AAU** | **178** | **1.78** | **113** | **1.67** | **65** | **2** |
|  | AAC | 22 | 0.22 | 22 | 0.33 | 0 | 0 |
| Lys (K) | **AAA** | **84** | **1.87** | **48** | **1.92** | **36** | **1.8** |
|  | AAG | 6 | 0.13 | 2 | 0.08 | 4 | 0.2 |
| Asp (D) | **GAU** | **56** | **1.72** | **38** | **1.65** | **18** | **1.89** |
|  | GAC | 9 | 0.28 | 8 | 0.35 | 1 | 0.11 |
| Glu (E) | **GAA** | **70** | **1.87** | **45** | **2** | **25** | **1.67** |
|  | GAG | 5 | 0.13 | 0 | 0 | 5 | 0.33 |
| Cys (C) | **UGU** | **31** | **1.82** | **10** | **1.82** | **21** | **1.83** |
|  | UGC | 3 | 0.18 | 1 | 0.18 | 2 | 0.17 |
| Trp (W) | **UGA** | **92** | **1.88** | **65** | **1.94** | **27** | **1.74** |
|  | UGG | 6 | 0.12 | 2 | 0.06 | 4 | 0.26 |
| Arg (R) | CGU | 13 | 0.9 | 6 | 0.63 | 7 | 1.4 |
|  | CGC | 0 | 0 | 0 | 0 | 0 | 0 |
|  | **CGA** | **40** | **2.76** | **31** | **3.26** | **9** | **1.8** |
|  | CGG | 5 | 0.34 | 1 | 0.11 | 4 | 0.8 |
| Ser^AGN^ (S) | AGU | 43 | 1.01 | 21 | 0.89 | 22 | 1.17 |
|  | AGC | 3 | 0.07 | 3 | 0.13 | 0 | 0 |
|  | **AGA** | **58** | **1.37** | **16** | **0.68** | **42** | **2.23** |
|  | AGG | 0 | 0 | 0 | 0 | 0 | 0 |
| Gly (G) | GGU | 48 | 0.87 | 24 | 0.72 | 24 | 1.1 |
|  | GGC | 2 | 0.04 | 2 | 0.06 | 0 | 0 |
|  | **GGA** | **132** | **2.39** | **92** | **2.75** | **40** | **1.84** |
|  | GGG | 39 | 0.71 | 16 | 0.48 | 23 | 1.06 |
