# Supplementary material for: The Phylogeny and Evolutionary Timescale of Muscoidea (Diptera: Brachycera: Calyptratae) Inferred from Mitochondrial Genomes
Source: PLoS One. 2015 Jul 30;10(7):e0134170. doi: 10.1371/journal.pone.0134170 (PMC4520480; doi:10.1371/journal.pone.0134170)
Supplement: S6 Table — (DOCX) [file pone.0134170.s008.docx]

**Table S6.** **Codon usage of the *Delia platura*. mt genome.**

| Amino acid | Codon | N | RSCU | N+ | RSCU | N- | RSCU |
| --- | --- | --- | --- | --- | --- | --- | --- |
| Phe (F) | **UUU** | **316** | **1.84** | **175** | **1.73** | **141** | **1.99** |
|  | UUC | 28 | 0.16 | 27 | 0.27 | 1 | 0.01 |
| Leu^UUR^ (L) | **UUA** | **520** | **5.16** | **281** | **4.92** | **239** | **5.47** |
|  | UUG | 15 | 0.15 | 3 | 0.05 | 12 | 0.27 |
| Leu^CUN^ (L) | **CUU** | **37** | **0.37** | **29** | **0.51** | **8** | **0.18** |
|  | CUC | 2 | 0.02 | 2 | 0.03 | 0 | 0 |
|  | CUA | 31 | 0.31 | 28 | 0.49 | 3 | 0.07 |
|  | CUG | 0 | 0 | 0 | 0 | 0 | 0 |
| Ile (I) | **AUU** | **342** | **1.9** | **226** | **1.86** | **116** | **1.98** |
|  | AUC | 18 | 0.1 | 17 | 0.14 | 1 | 0.02 |
| Met (M) | **AUA** | **212** | **1.83** | **117** | **1.9** | **95** | **1.74** |
|  | AUG | 20 | 0.17 | 6 | 0.1 | 14 | 0.26 |
| Val (V) | GUU | 72 | 1.49 | 37 | 1.23 | 35 | 1.92 |
|  | GUC | 2 | 0.04 | 2 | 0.07 | 0 | 0 |
|  | **GUA** | **113** | **2.34** | **77** | **2.57** | **36** | **1.97** |
|  | GUG | 6 | 0.12 | 4 | 0.13 | 2 | 0.11 |
| Ser^UCN^ (S) | **UCU** | **135** | **3.12** | **72** | **3.02** | **63** | **3.25** |
|  | UCC | 2 | 0.05 | 2 | 0.08 | 0 | 0 |
|  | UCA | 98 | 2.27 | 74 | 3.1 | 24 | 1.24 |
|  | UCG | 4 | 0.09 | 2 | 0.08 | 2 | 0.1 |
| Pro (P) | **CCU** | **74** | **2.21** | **54** | **2.16** | **20** | **2.35** |
|  | CCC | 5 | 0.15 | 4 | 0.16 | 1 | 0.12 |
|  | CCA | 50 | 1.49 | 39 | 1.56 | 11 | 1.29 |
|  | CCG | 5 | 0.15 | 3 | 0.12 | 2 | 0.24 |
| Thr (T) | **ACU** | **99** | **2.18** | **72** | **2.12** | **27** | **2.35** |
|  | ACC | 8 | 0.18 | 5 | 0.15 | 3 | 0.26 |
|  | ACA | 74 | 1.63 | 58 | 1.71 | 16 | 1.39 |
|  | ACG | 1 | 0.02 | 1 | 0.03 | 0 | 0 |
| Ala (A) | **GCU** | **107** | **2.47** | **65** | **2.28** | **42** | **2.85** |
|  | GCC | 14 | 0.32 | 13 | 0.46 | 1 | 0.07 |
|  | GCA | 49 | 1.13 | 35 | 1.23 | 14 | 0.95 |
|  | GCG | 3 | 0.07 | 1 | 0.04 | 2 | 0.14 |
| Tyr (Y) | **UAU** | **153** | **1.81** | **71** | **1.65** | **82** | **1.98** |
|  | UAC | 16 | 0.19 | 15 | 0.35 | 1 | 0.02 |
| Stop (*) | **UAA** | **9** | **1.8** | **7** | **1.75** | **2** | **2** |
|  | UAG | 1 | 0.2 | 1 | 0.25 | 0 | 0 |
| His (H) | **CAU** | **60** | **1.67** | **50** | **1.64** | **10** | **1.82** |
|  | CAC | 12 | 0.33 | 11 | 0.36 | 1 | 0.18 |
| Gln (Q) | **CAA** | **76** | **1.95** | **52** | **2** | **24** | **1.85** |
|  | CAG | 2 | 0.05 | 0 | 0 | 2 | 0.15 |
| Asn (N) | **AAU** | **181** | **1.84** | **112** | **1.76** | **69** | **1.97** |
|  | AAC | 16 | 0.16 | 15 | 0.24 | 1 | 0.03 |
| Lys (K) | **AAA** | **73** | **1.64** | **47** | **1.88** | **26** | **1.33** |
|  | AAG | 16 | 0.36 | 3 | 0.12 | 13 | 0.67 |
| Asp (D) | **GAU** | **57** | **1.7** | **38** | **1.62** | **19** | **1.9** |
|  | GAC | 10 | 0.3 | 9 | 0.38 | 1 | 0.1 |
| Glu (E) | **GAA** | **71** | **1.92** | **40** | **1.86** | **31** | **2** |
|  | GAG | 3 | 0.08 | 3 | 0.14 | 0 | 0 |
| Cys (C) | **UGU** | **33** | **1.94** | **11** | **1.83** | **22** | **2** |
|  | UGC | 1 | 0.06 | 1 | 0.17 | 0 | 0 |
| Trp (W) | **UGA** | **94** | **1.94** | **67** | **1.97** | **27** | **1.86** |
|  | UGG | 3 | 0.06 | 1 | 0.03 | 2 | 0.14 |
| Arg (R) | CGU | 6 | 0.43 | 2 | 0.22 | 4 | 0.8 |
|  | CGC | 0 | 0 | 0 | 0 | 0 | 0 |
|  | **CGA** | **45** | **3.21** | **34** | **3.78** | **11** | **2.2** |
|  | CGG | 5 | 0.36 | 0 | 0 | 5 | 1 |
| Ser^AGN^ (S) | AGU | 50 | 1.16 | 19 | 0.8 | 31 | 1.6 |
|  | AGC | 1 | 0.02 | 1 | 0.04 | 0 | 0 |
|  | **AGA** | **54** | **1.25** | **21** | **0.88** | **33** | **1.7** |
|  | AGG | 2 | 0.05 | 0 | 0 | 2 | 0.1 |
| Gly (G) | GGU | 60 | 1.09 | 31 | 0.91 | 29 | 1.38 |
|  | GGC | 4 | 0.07 | 4 | 0.12 | 0 | 0 |
|  | **GGA** | **127** | **2.31** | **90** | **2.65** | **37** | **1.76** |
|  | GGG | 29 | 0.53 | 11 | 0.32 | 18 | 0.86 |
